# Biglycan Is a Novel Mineralocorticoid Receptor Target Involved in Aldosterone/Salt-Induced Glomerular Injury

**DOI:** 10.3390/ijms23126680

**Published:** 2022-06-15

**Authors:** Toshifumi Nakamura, Benjamin Bonnard, Roberto Palacios-Ramirez, Amaya Fernández-Celis, Frédéric Jaisser, Natalia López-Andrés

**Affiliations:** 1 Centre de Recherche des Cordeliers, INSERM, Sorbonne Université, Université de Paris, 75006 Paris, France; bienvenu0607@gmail.com (T.N.); benjamin.bonnard@inserm.fr (B.B.); robertopalaciosramirez@gmail.com (R.P.-R.); 2 Cardiovascular Translational Research, Navarrabiomed (Miguel Servet Foundation), Instituto de Investigación Sanitaria de Navarra (IdiSNA), 31008 Pamplona, Spain; amaya.fernandez.decelis@navarra.es; 3 INSERM, Clinical Investigation Centre 1433, French-Clinical Research Infrastructure Network (F-CRIN) INI-CRCT (Cardiovascular and Renal Clinical Trialists), 54500 Nancy, France

**Keywords:** mineralocorticoid receptor, glomerular injury, biglycan, proteoglycan, toll-like receptor 4 (TLR4), C-C motif chemokine ligand 3 (CCL3)

## Abstract

The beneficial effects of mineralocorticoid receptor (MR) antagonists (MRAs) for various kidney diseases are established. However, the underlying mechanisms of kidney injury induced by MR activation remain to be elucidated. We recently reported aldosterone-induced enhancement of proteoglycan expression in mitral valve interstitial cells and its association with fibromyxomatous valvular disorder. As the expression of certain proteoglycans is elevated in several kidney diseases, we hypothesized that proteoglycans mediate kidney injury in the context of aldosterone/MR pathway activation. We evaluated the proteoglycan expression and tissue injury in the kidney and isolated glomeruli of uninephrectomy/aldosterone/salt (NAS) mice. The MRA eplerenone was administered to assess the role of the MR pathway. We investigated the direct effects of biglycan, one of the proteoglycans, on macrophages using isolated macrophages. The kidney samples from NAS-treated mice showed enhanced fibrosis and increased expression of biglycan accompanying glomerular macrophage infiltration and enhanced expression of TNF-α, iNOS, Nox2, CCL3 (C-C motif chemokine ligand 3), and phosphorylated NF-κB. Eplerenone blunted these changes. Purified biglycan stimulated macrophages to express TNF-α, iNOS, Nox2, and CCL3. This was prevented by a toll-like receptor 4 (TLR4) or NF-κB inhibitor, indicating that biglycan stimulation is dependent on the TLR4/NF-κB pathway. We identified the proteoglycan biglycan as a novel target of MR involved in MR-induced glomerular injury and macrophage infiltration via a biglycan/TLR4/NF-κB/CCL3 cascade.

## 1. Introduction

The mineralocorticoid receptor (MR), a ligand-activated transcription factor, is expressed by epithelial cells in the distal nephron of the kidney and in the epithelium of the intestine. The MR is activated by the steroid hormone aldosterone, as well as by corticosteroids, when the protecting enzyme 11b-hydroxysteroid dehydrogenase type 2 (HSD2) is not co-expressed. Its main function in the kidneys and colon is to regulate sodium balance and blood pressure [1,2,3]. Over the last two decades, several clinical trials have revealed the beneficial effects of MR antagonists (MRAs) in improving the prognosis of cardiovascular disease [4,5,6]. These findings have led to numerous studies aiming to identify the underlying mechanism by which MR activation in non-epithelial cells— cardiomyocytes, smooth muscle cells, endothelial cells, and myeloid cells, for example—is involved in cardiovascular diseases beyond the regulation of sodium homeostasis [7,8].

MR activation is also involved in kidney disease progression. Aldosterone/MR activation combined with high salt challenge was shown to induce kidney injury by accelerating inflammation, oxidative stress, and fibrosis in several preclinical studies [9,10,11,12]. The benefit of MRA in several kidney disorders, including diabetic nephropathy, chronic kidney disease (CKD), renal ischemia, and drug-induced renal injury, has been shown in preclinical and clinical studies [13]. Indeed, several studies using tissue-specific MR knockout mouse models have shown that the MR in various cell types, including smooth muscle cells, endothelial cells, and myeloid cells, contributes to the development of various kidney disorders, such as ischemic reperfusion- [14], cyclophosphamide- [15], and obesity-mediated kidney fibrosis [16], as well as glomerulonephritis [17]. However, the precise mechanisms by which MR activation causes kidney injury, with or without specific kidney disorders, is yet to be elucidated. Knowledge about the specific mechanisms underlying MR involvement in kidney disease will undoubtedly maximize the appropriate use of MRA for this pathological condition.

Recently, we reported a novel aldosterone/MR pathway involved in mitral valve disease. We showed that aldosterone stimulation leads to enhanced proteoglycan expression in mitral valve interstitial cells and that MRA treatment blunts the development of fibromyxomatous mitral valve and myocardial alterations associated with mitral valve prolapse in a mouse model [18,19]. Increased proteoglycan expression in the kidneys of human and animal samples has been reported [20,21,22,23] and MR antagonists have shown beneficial effects in several kidney diseases [13]. Therefore, upregulated proteoglycan may potentially play an essential role in aldosterone/MR pathway-induced kidney disorders.

Here, we hypothesized that MR activation in the kidney stimulates the expression of proteoglycans known to be involved in kidney injury, including inflammation, fibrosis, and oxidative stress. We evaluated the alteration and localization of proteoglycans expression in the kidney in the uninephrectomy/aldosterone/salt model and explored whether modulated proteoglycans are associated with renal injury.

## 2. Results

### 2.1. NAS Treatment-Induced Kidney Injury Is Blunted by Eplerenone

As expected, mice treated by NAS challenge for six weeks showed higher systolic blood pressure, plasma creatinine levels, urinary albumin levels, and kidney weight than control mice (Table 1). The administration of the MRA eplerenone at a daily dose of 100 mg/kg blunted the above effects, except for the elevation of systolic blood pressure.

**Figure 1:**
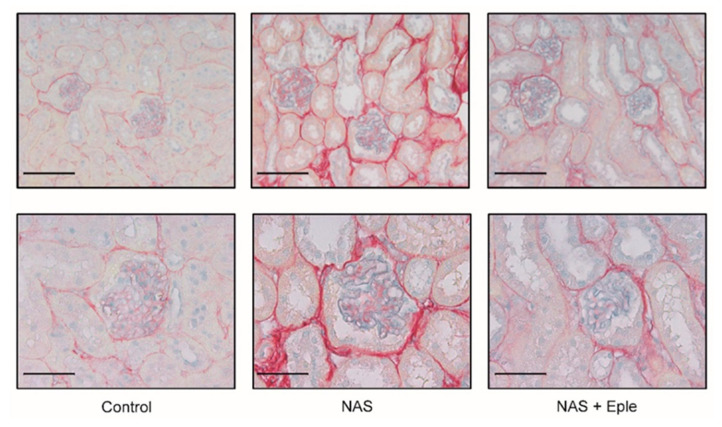
NAS treatment-induced renal fibrosis and proteoglycan accumulation are blunted by eplerenone. Representative microphotographs of mouse kidney sections with Sirius Red/Alcian blue staining: 20× (scale bar = 100 µm) and 40× (scale bar = 50 µm) magnification. NAS, uninephrectomy/aldosterone/salt; Eple, eplerenone.

Sirius Red staining of the kidney sections showed increased tubulointerstitial fibrosis staining (Figure 1) and the accumulation of thick and thin collagen fibers (Figure S1A). Renal gene expression of transforming growth factor-β1 (TGF-β1) and collagen type 1 and type 3 increased upon NAS treatment (Figure S1B). These changes were blunted by eplerenone treatment (Figure 1 and Figure S1A,B).

### 2.2. Proteoglycans Accumulate in NAS-Treated Kidneys

**Figure 2:**
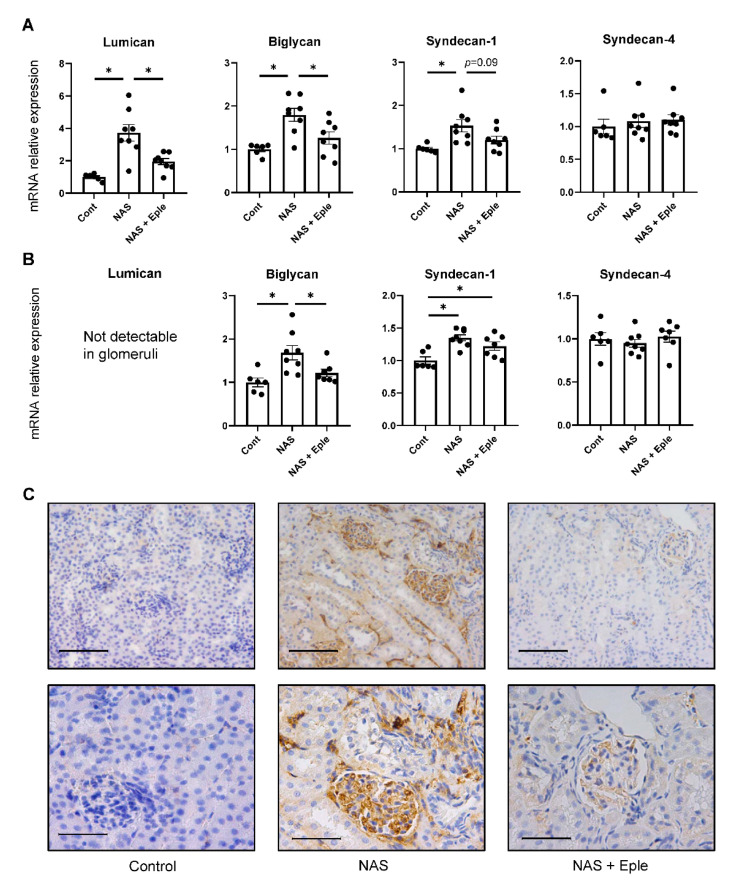
Proteoglycan accumulates in the kidney following NAS treatment. Proteoglycan mRNA levels in the kidney cortex (**A**) and isolated glomeruli (**B**). (**C**) Representative microphotographs of mouse kidney sections with immunostaining for biglycan. One-way ANOVA was used for statistical analysis, *n* = 6–8. * *p* < 0.05. 20× (scale bar = 100 µm) and 40× (scale bar = 50 µm) magnification. NAS, uninephrectomy/aldosterone/salt; Eple, eplerenone.

Alcian blue staining is commonly used to visualize proteoglycans. Upon NAS challenge, Alcian blue staining showed increased proteoglycan expression, especially in glomeruli (Figure 1). We evaluated the mRNA levels of several proteoglycans, including lumican, biglycan, syndecan-1, and syndecan-4, in kidney cortex samples (Figure 2A). Lumican and biglycan showed enhanced expression in the NAS-treated group, which was blunted in the eplerenone-treated group, whereas the upregulation of syndecan-1 was not modified by eplerenone. Expression of syndecan-4 was not modulated. We next identified the proteoglycan(s) for which the levels increased in the glomeruli showing intense positive Alcian-blue staining by isolating glomeruli for each treatment group. Lumican expression was not detectable in the glomeruli (Figure 2B) and syndecan-4 expression was unchanged. However, mRNA levels of biglycan and syndecan-1 were higher in the glomeruli of NAS-treated mice and eplerenone only blunted the increase in those of biglycan (Figure 2B).

As only biglycan expression was modulated by NAS treatment and blunted by eplerenone in both the kidney cortex and glomeruli, we focused on biglycan. Immunoblotting performed on the kidney cortex extract showed increased biglycan expression in the NAS-treated group, which was blunted in the eplerenone group, in accordance with the changes in mRNA levels (Figure S2A,B). We next assessed the renal expression of biglycan immunostaining (Figure 2C). No biglycan staining was detectable, either in the tubulointerstitium or glomeruli of control mice. Consistent with the results of the real-time qPCR evaluation, NAS treatment significantly increased biglycan deposition in the interstitium (in particular, in the glomeruli), which was blunted by eplerenone (Figure 2C).

### 2.3. NAS Treatment Accelerates Macrophage Infiltration of the Kidney, Especially of the Glomeruli

**Figure 3:**
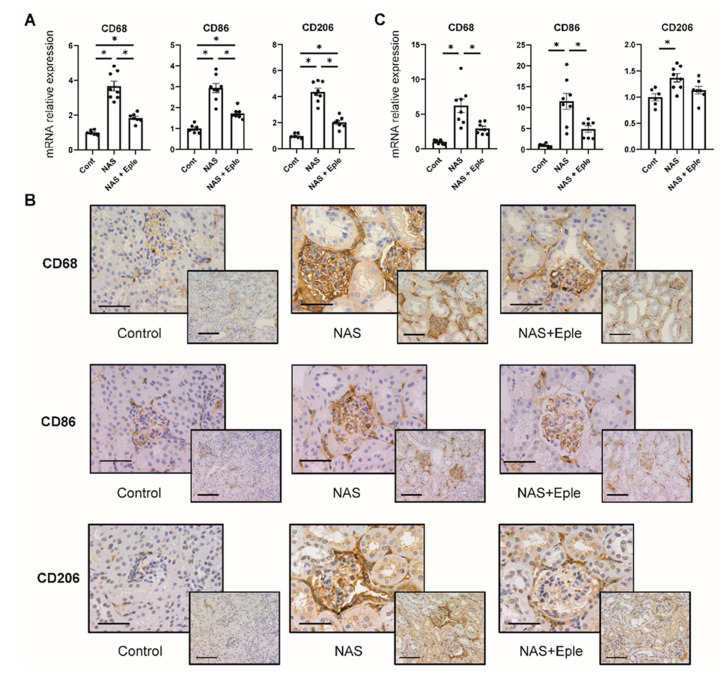
NAS treatment accelerates macrophage infiltration in the kidney, especially in the glomeruli. Macrophage marker (CD68, CD86, and CD206) mRNA levels in the kidney cortex (**A**) and isolated glomeruli (**C**). (**B**) Representative microphotographs of mouse kidney sections with immunostaining for macrophage markers (CD68, CD86, and CD206). One-way ANOVA was used for statistical analysis, *n* = 6–8. * *p* < 0.05. 20× (scale bar = 100 µm) and 40× (scale bar = 50 µm) magnification. NAS, uninephrectomy/aldosterone/salt; Eple, eplerenone; CD68, cluster of differentiation 68; CD86, cluster of differentiation 86; CD206, cluster of differentiation 206.

Macrophage infiltration of the kidney has been reported to be increased by either NAS treatment [9] or in vivo biglycan overexpression [24]. The mRNA levels of CD68, a pan-macrophage marker, were up-regulated in the kidney cortex (Figure 3A). Consistent with this result, CD68 immunostaining was more intense in the NAS-treated group, especially in the glomeruli (Figure 3B). To further characterize these macrophages, we evaluated mRNA expression and immunostaining for CD86, as an M1 marker, and CD206, as an M2 marker. NAS treatment increased both the mRNA levels and immunostaining of these two markers in the kidney. This was blunted by the MRA eplerenone (Figure 3A,B). As the glomeruli were markedly stained in the NAS-treated group (Figure 3B), we analyzed the mRNA expression of these markers in isolated glomeruli: gene expression of CD68, CD86, and CD206 increased in the NAS-treated group by 6.2-fold, 11.5-fold, and 1.4-fold, respectively (Figure 3C). Eplerenone blunted the increased expression of CD68 and CD86 in the glomeruli (Figure 3B,C), whereas the expression of CD206 was not significantly blunted.

### 2.4. NAS Treatment Enhances the Expression of the Chemoattractant CCL3

**Figure 4:**
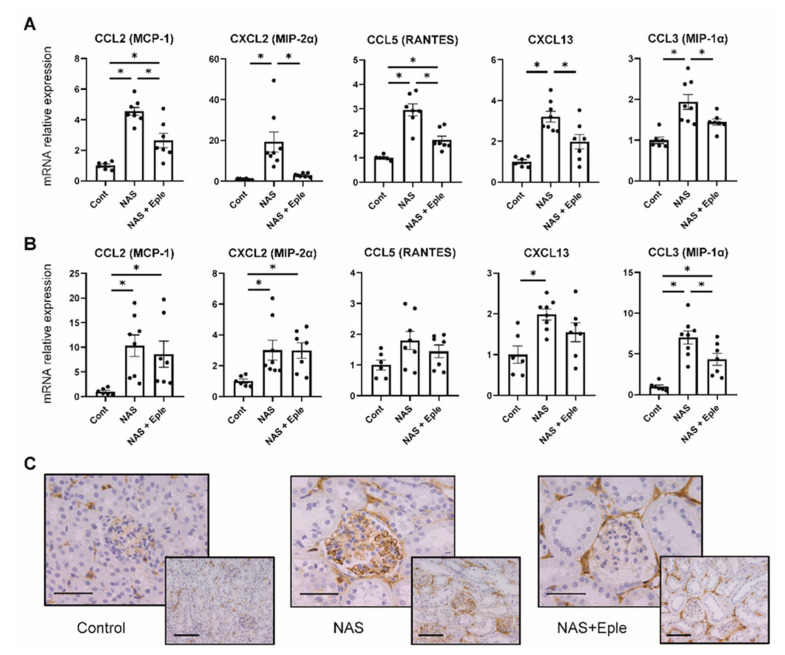
NAS treatment enhances the expression of chemoattractants. Macrophage chemoattractant (CCL2, CXCL2, CCL5, CXCL13, and CCL3) mRNA levels in the kidney cortex (**A**) and isolated glomeruli (**B**). (**C**) Representative microphotographs of mouse kidney sections with immunostaining for CCL3. One-way ANOVA was used for statistical analysis, *n* = 6–8. * *p* < 0.05. 20× (scale bar = 100 µm) and 40× (scale bar = 50 µm) magnification. NAS, uninephrectomy/aldosterone/salt; Eple, eplerenone; CCL2, C-C motif chemokine ligand 2, MCP-1, monocyte chemoattractant protein 1; CCL3, C-C motif chemokine ligand 3; MIP-1α, macrophage inflammatory protein-1α; CCL5, C-C motif chemokine ligand 5; RANTES, regulated upon activation, normally T-expressed, and presumably secreted; CXCL2, C-X-C motif chemokine ligand 2; MIP-2α, macrophage inflammatory protein 2α; CXCL13, C-X-C motif chemokine ligand 13.

We next evaluated the underlying mechanism of enhanced macrophage infiltration by measuring the gene expression of several chemoattractant chemokines known to be modulated by biglycan [24,25], including CCL2 (MCP-1), CXCL2 (MIP-2α), CCL5 (RANTES), CXCL13, and CCL3 (MIP-1α). In the kidney cortex, NAS treatment upregulated the expression of all chemokines and eplerenone treatment blunted their expression (Figure 4A). In the glomeruli of the NAS-treated group, we observed increased expression of most of these chemoattractants except CCL5, but eplerenone significantly blunted the observed increase solely for CCL3 (Figure 4B). The upregulation of CCL3 in the glomeruli of the NAS-treated group and its prevention by eplerenone was confirmed by CCL3 immunostaining (Figure 4C).

### 2.5. TLR4 Pathway Activation Is Modulated by NAS and Blunted by the MRA Eplerenone

**Figure 5:**
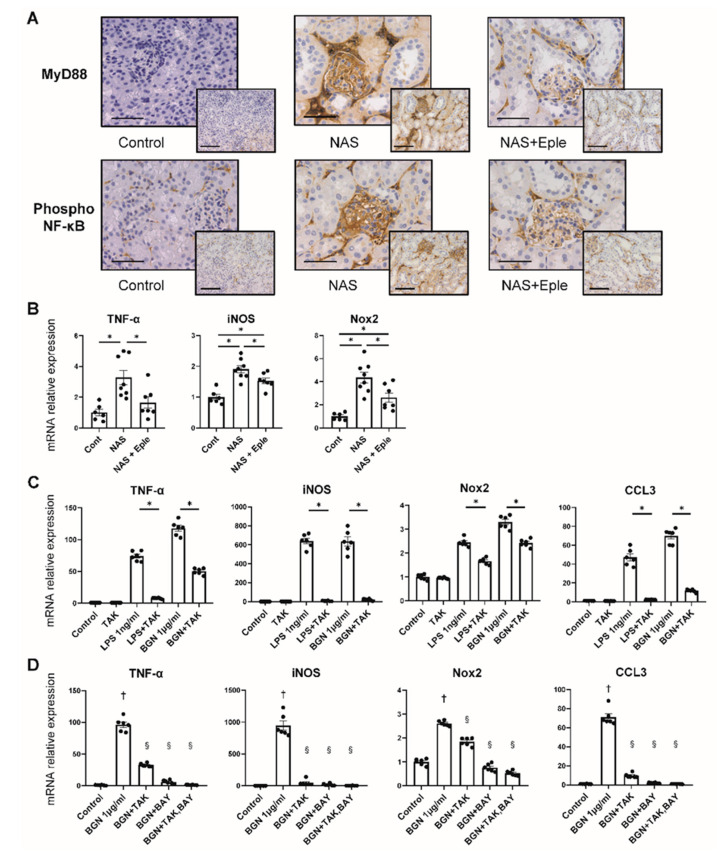
TLR4 pathway activation is modulated by NAS and blunted by eplerenone. (**A**) Representative microphotographs of mouse kidney sections with immunostaining for MyD88 and phosphorylated NF-κB. 20× (scale bar = 100 µm) and 40× (scale bar = 50 µm) magnification. (**B**) TNF-α, iNOS, and Nox2 mRNA levels in isolated glomeruli. (**C**,**D**) TNF-α, iNOS, Nox2, and CCL3 mRNA levels in isolated peritoneal macrophages. LPS and biglycan stimulated isolated peritoneal macrophages to express these genes, which was prevented by TAK-242 (**C**). TAK-242 and BAY11-7082, a NF-κB inhibitor, prevented biglycan-induced expression of TNF-α, iNOS, Nox2, and CCL3 (**D**). One-way ANOVA was used for statistical analysis, *n* = 6–8 for isolated glomeruli, *n* = 6 for isolated peritoneal macrophages, * *p* < 0.05. ^†^ *p* < 0.05 vs. control, ^§^ *p* < 0.05 vs. biglycan. NAS, uninephrectomy/aldosterone/salt; Eple, eplerenone; TNF-α, tumor necrosis factor-α; iNOS, inducible nitric oxide synthase; Nox2, NADPH oxidase 2; CCL3, C-C motif chemokine ligand 3; LPS, lipopolysaccharide; BGN, biglycan; TAK, TAK-242; BAY, BAY11-7082; TLR4, toll-like receptor.

Biglycan is known to trigger inflammatory signaling: biglycan acts as a ligand of toll-like receptor 4 (TLR4) expressed on macrophages and activates the TLR4 downstream signaling pathway [20]. We therefore hypothesized that the TLR4 pathway could be activated in the glomeruli of NAS-treated mice where enhanced biglycan expression was observed. We evaluated the activation of this pathway in glomeruli by analyzing the expression of MyD88 and NF-κB, the mediators of TLR4 signaling, by immunostaining. MyD88 and phosphorylated NF-κB, an active form of NF-κB, were intensely positive in the NAS-treated group, mostly in the glomeruli, whereas eplerenone blunted this effect (Figure 5A). Next, we analyzed the gene expression of TNF-α, iNOS, and Nox2, which are known to be the downstream targets of TLR4 signaling, in isolated glomeruli. The mRNA levels of these genes increased after NAS treatment and the effect was blunted by eplerenone treatment (Figure 5B).

### 2.6. The Stimulation of CCL3 Expression by Biglycan Is TLR4 Pathway-Dependent in Isolated Macrophages

We observed activation of the TLR4 pathway and enhanced expression of CCL3, together with increased glomerular macrophage infiltration, in glomeruli upon NAS treatment. We thus explored the impact of purified biglycan on macrophages and the role of the TLR4 pathway. As reported previously, LPS, a TLR4 agonist, stimulated the macrophages to increase the expression of TNF-α, iNOS, Nox2, and CCL3, which was prevented by TLR4 inhibitor treatment (Figure 5C). Next, we treated the isolated macrophages with purified biglycan. This treatment resulted in an increase in the gene expression of TNF-α, iNOS, Nox2, and CCL3 to the same extent as with LPS treatment (Figure 5C). The effect of purified biglycan was dose-dependent (Figure S3). Co-treatment with a TLR4 inhibitor (TAK-242) and/or NF-κB inhibitor (BAY11-7082) prevented the increase in the expression of TNF-α, iNOS, Nox2, and CCL3. These data show that biglycan-induced expression of the macrophage chemoattractant chemokine CCL3 is dependent on the TLR4/NF-κB pathway.

## 3. Discussion

Our current experiments confirmed that MR activation through NAS treatment results in enhanced renal fibrosis and inflammation and the MRA eplerenone blunted these effects. Notably, eplerenone did not significantly prevent the elevation of the plasma creatinine level induced by NAS treatment, despite the decrease of macrophage infiltration and of urinary albumin level. This is consistent with previous studies [26,27] and may be related to the effect of MRAs preventing glomerular hyperfiltration due to aldosterone excess [28].

We identified the proteoglycan biglycan as a novel MR-modulated target in the kidney, especially in the glomeruli. Notably, macrophage infiltration was prominent in the glomeruli in NAS-treated mice, accompanied by enhanced expression of biglycan and the chemoattractant chemokine CCL3. The staining of MyD88 and phosphorylated NF-κB suggests activation of the TLR4/MyD88/NF-κB pathway and is consistent with the enhanced expression of the downstream TLR4 targets TNF-α, iNOS, and Nox2. These changes, confirmed in NAS-treated mice, were prevented by the MRA eplerenone, indicating a novel target of pharmacological MR antagonism upon renal injury. Cellular experiments demonstrated the direct effect of biglycan in stimulating macrophages to express CCL3 in a TLR4/NF-κB pathway-dependent manner.

To date, the capacity of aldosterone/salt treatment to induce kidney injury through enhanced inflammation, oxidative stress, and fibrosis has been well-documented [13]. Several studies have demonstrated that aldosterone/salt treatment induces inflammatory cytokine expression, including that of IL-1β, IL-6, and CCL2, in the kidney [9,29,30]. Furthermore, flow cytometry analysis showed enhanced infiltration of M1 type macrophages, accompanied by increased TNF-α expression, in the kidneys of aldosterone/salt-treated rats [12]. Spironolactone treatment improved these features [12]. Other studies have shown that aldosterone/salt treatment increases the expression of NADPH oxidase subunits in the rat kidney, which is blunted by eplerenone treatment [31,32]. The specific mechanism linking MR activation from the aldosterone/salt challenge and macrophage recruitment and stimulation in the kidney is yet to be explored. Numerous studies have also demonstrated that MR activation with aldosterone/salt treatment causes consequent renal fibrosis and extracellular matrix (ECM) accumulation, with increased expression of collagen and fibronectin [9,10,29,33]. However, the modulation of proteoglycans, which are also part of the ECM, has not been assessed in this model. We recently demonstrated that aldosterone increases the expression of several proteoglycans, such as biglycan, lumican, and syndecan-1, in cardiac valvular cells, suggesting a link between MR signaling and proteoglycan expression in cardiac valvular degeneration [18,19]. The current study shows that the expression of biglycan and lumican are modulated by NAS and eplerenone treatment in the kidney. Interestingly, biglycan was also modulated in glomeruli, suggesting a specific role for biglycan in MR-mediated glomerular injury. Whether biglycan is directly or indirectly modulated upon MR activation is yet to be studied. TGF-β stimulation of various renal cell types, including mesangial cells, increases biglycan expression [34,35], and TGF-β is a well-known downstream target of MR activation (as we also observed in the present study). Therefore, TGF-β could be an intermediate between MR and biglycan expression. Overall, the current study suggests that enhanced biglycan expression in glomeruli may have a significant role in aldosterone/MR-associated kidney injury.

Proteoglycans are glycosylated molecules in which glycosaminoglycan (GAG) is attached to a core protein. GAG is generally sulfated, and the chain length and position of sulfation varies between each proteoglycan. Such variation gives each proteoglycan distinct functional features [36]. Proteoglycans have been reported to accumulate in areas of tubulointerstitial fibrosis and glomeruli in several experimental models and in human kidney disease [22,23,37]. Biglycan is one of several small, leucine-rich proteoglycans and is expressed mainly in the interstitium in the normal kidney. Under normal conditions, biglycan is sequestered in the ECM and interacts via its core protein or GAG chains with other components of the ECM, including collagen types I, II, III, and VI and elastin [38]. GAGs can interact with many bioactive binding partners to trigger cell signaling, proliferation, and ECM production. Over the last two decades, biglycan has been shown to trigger an inflammatory cascade and to be a ligand of TLR4 expressed in macrophages [20]. *In vivo*, biglycan overexpression increases renal leukocyte infiltration, mainly that of macrophages and T cells [24,25]. Overexpressed biglycan also induces renal expression of several inflammatory cytokines and chemoattractants, including TNF-α, IL-1β, CCL2, CCL3, CCL5, CXCL2, and CXCL13 in vivo [24,25]. Interestingly, increased expression of these cytokines and chemoattractants upon biglycan overexpression was shown to be blunted in TLR4-deleted mice, suggesting that biglycan induces chemoattractant expression and leukocyte recruitment through the TLR4 pathway [24,25]. Another study showed that renal ischemia-reperfusion injury (IRI) is accompanied by increased expression of biglycan and chemoattractants (CCL2, CCL5, and CXCL1) [39]. Enhanced leukocyte infiltration and expression of chemoattractant chemokines after IRI were also prevented in mice with TLR2 and TLR4 deletion [39]. Although the IRI nephropathy model is different from the aldosterone/salt model, these studies showed a significant role for increased biglycan expression in the progression of kidney injury.

In accordance with these studies, our data showed enhanced macrophage infiltration and upregulated expression of biglycan and several chemoattractants in the NAS-treated group and the beneficial effect of the MRA eplerenone. In NAS-treated mice, we observed positive staining of phosphorylated NF-κB and upregulation of TLR4 downstream targets, suggesting TLR4 pathway activation in glomeruli. Moreover, we observed enhanced expression of the chemokine CCL3 (also known as MIP-1α) as a novel MR downstream target in the glomeruli. Eplerenone treatment blunted these alterations, indicating that this could contribute to the beneficial effects of MR antagonism in the kidney.

CCL3 is among the chemoattractant chemokines produced by monocytes, macrophages, and lymphocytes [40]. CCL3 is known to interact with CCR1, mainly expressed on macrophages and T cells, and to initiate an inflammatory response that leads to the recruitment of these cells [41]. Several studies have reported the involvement of CCL3 in kidney diseases. CCR1 deletion or specific CCR1 antagonism blunted the increased infiltration of macrophages and neutrophils observed in renal IRI and in a lupus-prone mouse model [42,43]. Although the association of CCL3 with aldosterone/salt-induced nephropathy has not been studied to date, these previous studies suggest a significant role for CCL3 in the pathogeny of kidney damage. Activation of the TLR4 pathway has been reported to induce CCL3 expression in macrophages, and anti-TLR4 antibody treatment or MyD88 deletion was shown to prevent such induction in human monocytic cells [44]. Taken together with the *in vivo* studies showing that biglycan overexpression induces the expression of inflammatory cytokines, including CCL3 [24,25], we propose a potential role for biglycan in inducing CCL3 expression through the TLR4 pathway. Indeed, in our study, biglycan stimulated isolated macrophages to express CCL3, which was fully prevented by TLR4 inhibition, showing biglycan to be a modulator of CCL3 expression through the TLR4 pathway. Interestingly, all the genes modulated by NAS and eplerenone treatment in the glomeruli in the current study, such as TNF-α, iNOS, and Nox2, were also regulated by the biglycan/TLR4/NF-κB signaling pathway. Although the pathophysiological involvement of TLR4 activation in vivo was not assessed in the present study, pharmacological TLR4 inhibition has been reported to blunt NAS-induced renal and cardiac injury in rats [45]. Overall, our findings suggest a possible role for CCL3 as a novel actor downstream of MR signaling through biglycan expression and the TLR4 pathway, leading to macrophage infiltration and activation in aldosterone/salt-induced kidney injury.

In conclusion, this study identifies biglycan as a possible novel target modulated by an MRA in glomeruli upon NAS-induced kidney injury. The increased CCL3 expression and M1 type macrophage infiltration and activation in glomeruli following downstream activation of the TLR4 pathway suggests a novel mechanism for MR-mediated kidney injury via a biglycan-TLR4-CCL3-macrophage cascade (Figure S4). Further studies are warranted to link these observations to functional alterations, such as albuminuria or renal failure, and determine how this contributes to the reported benefits of MR antagonism in kidney diseases.

## 4. Materials and Methods

### 4.1. Mice

**Figure 6:**
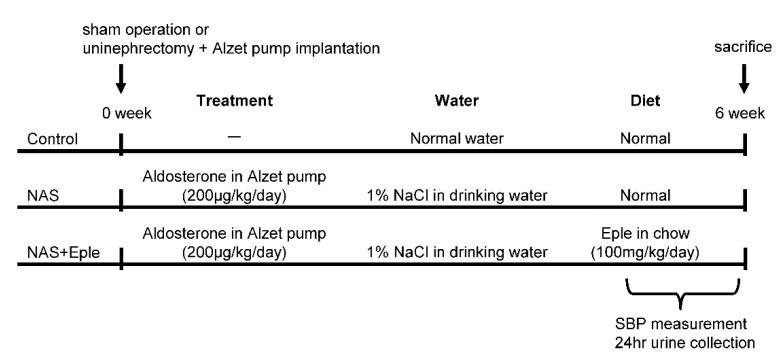
Scheme of experimental protocol. NAS, uninephrectomy/aldosterone/salt; Eple, eplerenone; SBP, systolic blood pressure.

All animal studies were conducted in accordance with the National Institutes of Health Guide and European Community Directives for the Care and Use of Laboratory Animals (European Directive, 2010/63/UE), approved by the local animal ethics committee (APAFIS#7420-2016102718076839), and conducted according to the INSERM animal care and use committee guidelines. The animals were housed in controlled-climate conditions under a 12 h light/dark cycle and allowed free access to food and water. Male C57Bl/6 mice (Charles River Laboratories, Les Oncins, France) were grouped as sham-treated control, nephrectomy-aldosterone-salt (NAS) treatment, and NAS with MRA treatment (Figure 6). For the NAS treatment, mice were uninephrectomized and osmotic minipumps (Alzet, Charles River) containing aldosterone (200 μg/kg per day; Sigma-Aldrich, St. Louis, MO, USA) were implanted subcutaneously under ketamine/xylazine anesthesia. The day after surgery, the drinking water was replaced with 1% NaCl. For MRA treatment, eplerenone (homogenized tablets, INSPRA, Pfizer, France) was orally administered (100 mg/kg/day, mixed into the chow) during the six-weeks of NAS treatment. In sham-treated animals, the kidney was exposed but not removed. Systolic blood pressure was measured using tail cuff plethysmography (BP2000; Bioseb, Vitrolles, France) of trained conscious mice according to the manufacturer’s manual. The mean of ten measurements for each mouse was determined every day for five days. To collect urine, all mice were individually placed in mouse metabolic cages for 24 h. The urinary albumin concentration was measured by ELISA according to the manufacturer’s instructions (Cristal Chem, Chicago, IL, USA).

### 4.2. Tissue Collection

At the end of the experiment, blood samples were collected and plasma creatinine and urea concentrations determined using an automatic analyzer (Konelab 20i; Thermo Fisher Scientific, Vernon Hills, IL, USA). The kidney was dissected and divided into three parts. One was immersion-fixed in 10% buffered formalin for histological studies, another snap-frozen for molecular analysis, and the last used for glomeruli isolation, as described below.

### 4.3. Glomeruli Isolation

Mouse glomeruli were isolated as previously described [46]. Briefly, minced mouse kidneys were incubated with 1mg/mL collagenase A (Sigma-Aldrich, St. Louis, MO, USA) in HBSS (Gibco, Grand Island, NY, USA) buffer at 37 °C for 30 min with pipetting at 10 min intervals. After centrifugation, the digested tissue was resuspended in HBSS. The tissue mixture was passed through 100 and 70 µm strainers (BD Falcon, Franklin lakes, NJ, USA). The filtrate, including glomeruli and tubular fragments, was collected using a 40 µm strainer. The retained fragments were transferred into a cell-culture dish. After 1 to 2 min of settling, tubular fragments adhered to the bottom of the dish. The glomeruli-enriched supernatant was transferred onto another 40 µm strainer and the settling procedure repeated to remove residual tubular fragments. The supernatant containing highly purified glomeruli was collected and subjected to the subsequent analyses.

### 4.4. Peritoneal Macrophage Isolation and Treatment

Peritoneal macrophages (PMs) were isolated as previously described [47]. Briefly, male C57Bl/6 mice were injected intraperitoneally with 1 mL 3% brewer thioglycolate medium (Sigma-Aldrich). Four days later, PMs were obtained via peritoneal lavage with DPBS and resuspended in RPMI 1640 (Gibco) supplemented with 1% penicillin and streptomycin and 10% charcoal-stripped fetal bovine serum. PMs were plated and left to adhere for 2 h at 37 °C in a 5% CO2 atmosphere before being washed with warm DPBS. The next day, PMs were treated with 1 ng/mL lipopolysaccharide (LPS) or 0.2, 1, or 5 µg/mL biglycan for 4 h. To explore the underlying mechanisms of the effect of biglycan, the toll-like receptor 4 inhibitor (TLR4i) TAK-242 (Sigma-Aldrich) or the NF-kB inhibitor BAY 11-7082 (Sigma-Aldrich) was administered 1 h prior to treatment with 1.0 µg/mL biglycan.

### 4.5. RNA Extraction and Real-Time Quantitative PCR

Total RNA was extracted from kidney samples and cells using TRI reagent (Sigma-Aldrich) according to the manufacturer’s instructions. Total RNA was reverse transcribed into cDNA using an M-MLV reverse transcriptase kit (Invitrogen, Carlsbad, CA, USA) and quantitative PCR carried out using iQ SYBR Green Supermix (Bio-Rad, Hercules, CA, USA) on a CFX384 Real Time thermal cycler (Bio-Rad). The target genes were as follows: CCL2 (C-C motif chemokine ligand 2, also known as monocyte chemoattractant protein 1: MCP-1), CCL3 (C-C motif chemokine ligand 3, also known as macrophage inflammatory protein-1α: MIP-1α), CCL5 (C-C motif chemokine ligand 5, also known as regulated upon activation, normally T-expressed, and presumably secreted: RANTES), CXCL2 (C-X-C motif chemokine ligand 2, also known as macrophage inflammatory protein 2α: MIP-2α), CXCL13 (C-X-C motif chemokine ligand 13), TNF-α (tumor necrosis factor-α), iNOS (inducible nitric oxide synthase), Nox2 (NADPH oxidase 2), collagen types 1a1 and 3a1, TGF-β1 (transforming growth factor β1), CD68 (cluster of differentiation 68), CD86, and CD206. Target gene expression was normalized to GAPDH (glyceraldehyde 3-phosphate dehydrogenase) mRNA expression. Primer sequences are shown in Table S1.

### 4.6. Immunoblotting

The kidney samples were homogenized using Roche cOmplete lysis-M buffer with a protease inhibitor and phosphatase inhibitor cocktail (Roche, Neuilly, France). Extracts were centrifuged and the protein concentration in the supernatant determined by the Lowry method according to the manufacturer’s instructions. Aliquots of 10 to 15 µg of total protein were prepared from the renal cortex, electrophoresed on SDS polyacrylamide gels, and transferred to Hybond-c Extra nitrocellulose membranes (Bio-Rad). Membranes were incubated with primary antibodies for biglycan (1:500, Santa Cruz Biotechnology, Santa Cruz, CA, USA). Stain-free detection and β-actin (Sigma-Aldrich) were used as loading controls. After washing, the membranes were incubated in peroxidase-conjugated secondary antibody and the target proteins detected using an ECL chemiluminiscence kit (Amersham). Band densitometry was performed using Image Lab software (Bio-Rad) and the results are expressed in arbitrary units (AU). All Western blots were performed at least in triplicate for each experimental condition.

### 4.7. Immunohistological Evaluation

Histological evaluation of kidney samples was performed on 5 μm thick paraffin-embedded serial sections following the protocol of the Leica BOND-Polymer Re-fine Detection automatic immunostainer (Leica, Wetzlar, Germany). All solutions were poured into the bottle-Bond Open Container (Leica) and registered on a computer using the Leica Biosystem program. The immunostaining program protocol included a fixative solution, a bond wash solution, blocking with a common immunohistochemistry blocker, and incubation with the primary antibody for biglycan (Santa Cruz Biotechnology), CD68 (Abcam, Cambridge, MA), CD86 (Santa Cruz Biotechnology), CD206 (Santa Cruz Biotechnology), CCL3 (Santa Cruz Biotechnology), MyD88 (myeloid differentiation factor 88, Santa Cruz Biotechnology), and phospho NF-κB (Cell Signaling). After primary antibody incubation, slides were incubated with secondary poly-HRP-IgG. The signal was revealed using DAB substrate. Incubation without a primary antibody was carried out as a negative control.

All tissues were hydrated in water. For proteoglycan and collagen identification, slides were incubated with Alcian blue (Sigma-Aldrich) for 20 min and then with 1% Sirius Red in picric acid for 30 min. Images from histological and immunohistochemistry preparations were captured using bright field or polarized light, as appropriate, with an automated image analysis system (Nikon, Tokyo, Japan). The most representative images are shown in the figures.

### 4.8. Statistical Analysis

Data are presented as the mean ± SEM. Differences in baseline characteristics, mRNA expression, and protein expression between groups were analyzed with one-way analysis of variance (ANOVA) followed by Tukey multiple comparisons tests. Statistical analysis was performed using GraphPad Prism version 8.0 (GraphPad Software, San Diego, CA, USA). We considered *p*-values < 0.05 to be statistically significant.

## Figures and Tables

**Table 1:** Characteristics of the treated mice.

	Control (*n* = 6)	NAS (*n* = 8)	NAS + Eple (*n* = 8)
Body weight (g)	24.6 ± 0.7	24.6 ± 0.3	24.1 ± 0.4
Blood pressure (mmHg)	119.5 ± 2.5	137.2 ± 3.3 **	134.7 ± 3.6 **
Plasma creatinine (μM)	10.35 ± 0.51	19.54 ± 1.39 **	16.41 ± 1.65 *
Plasma urea (mM)	9.05 ± 0.43	8.69 ± 0.58	9.10 ± 0.34
Urinary albumin (µg/24 h)	12.0 ± 0.8	116.0 ± 27.5 **	45.5 ± 9.5 ^†^
Kidney weight/tibia length (mg/mm)	8.56 ± 0.34	18.37 ± 0.82 **	15.00 ± 0.68 **^‡^

Data are presented as the mean ± SEM. * *p* < 0.05 vs. Control, ** *p* < 0.01 vs. Control, ^†^ *p* < 0.05 vs. NAS, ^‡^ *p* < 0.01 vs. NAS. NAS, uninephrectomy/aldosterone/salt., Eple, eplerenone.

## Data Availability

Not applicable.

## Supplementary Materials

The following supporting information can be downloaded at: https://www.mdpi.com/article/10.3390/ijms23126680/s1.
