# Supplementary material for: Biglycan Is a Novel Mineralocorticoid Receptor Target Involved in Aldosterone/Salt-Induced Glomerular Injury
Source: Int J Mol Sci. 2022 Jun 15;23(12):6680. doi: 10.3390/ijms23126680 (PMC9224513; doi:10.3390/ijms23126680)
Supplement: Supplementary file 1 [file ijms-23-06680-s001.zip › ijms-1750486-supplementary.pdf]

Supplemental Material

Biglycan is a novel mineralocorticoid receptor target involved in aldosterone/salt-induced glomerular injury

Toshifumi Nakamura[1], Benjamin Bonnard[1], Roberto Palacios-Ramirez[1], Amaya Fernández-Celis[2], Frédéric Jaisser[1,3], and Natalia López-Andrés[2].

[1] Centre de Recherche des Cordeliers, INSERM, Sorbonne Université, Université de Paris, 75006 Paris, France.

[2] Cardiovascular Translational Research, Navarrabiomed (Miguel Servet Foundation), Instituto de Investigación Sanitaria de Navarra (IdiSNA), Pamplona, Spain.

[3] INSERM, Clinical Investigation Centre 1433, French-Clinical Research Infrastructure Network (F-CRIN) INI-CRCT (Cardiovascular and Renal Clinical Trialists), Nancy, France.

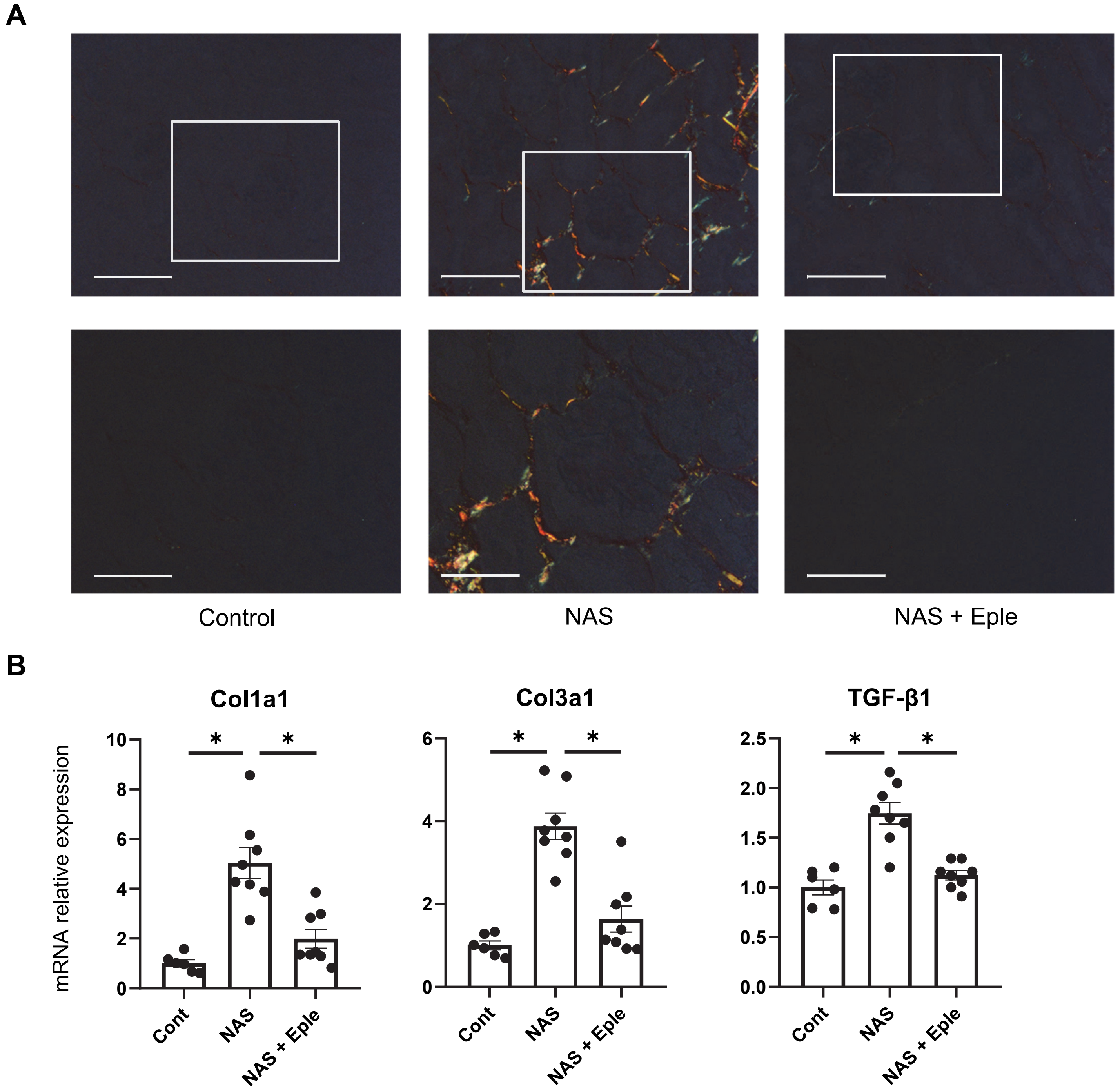

Figure S1. NAS treatment-induced renal fibrosis is blunted by eplerenone. Representative microphotographs of mouse kidney sections with Sirius red observed by polarized light microscopy (A). Profibrotic gene mRNA levels (B). 20× (scale bar = 100 μm) and 40× (scale bar = 50 μm) magnification. One-way ANOVA was used for statistical analysis, n = 6–8. *P < 0.05. NAS, uninephrectomy/aldosterone/salt; Eple, eplerenone; Col1a1, collagen type 1a1; Col3a1, collagen type 3a1; TGF-β1, transforming growth factor-β1.

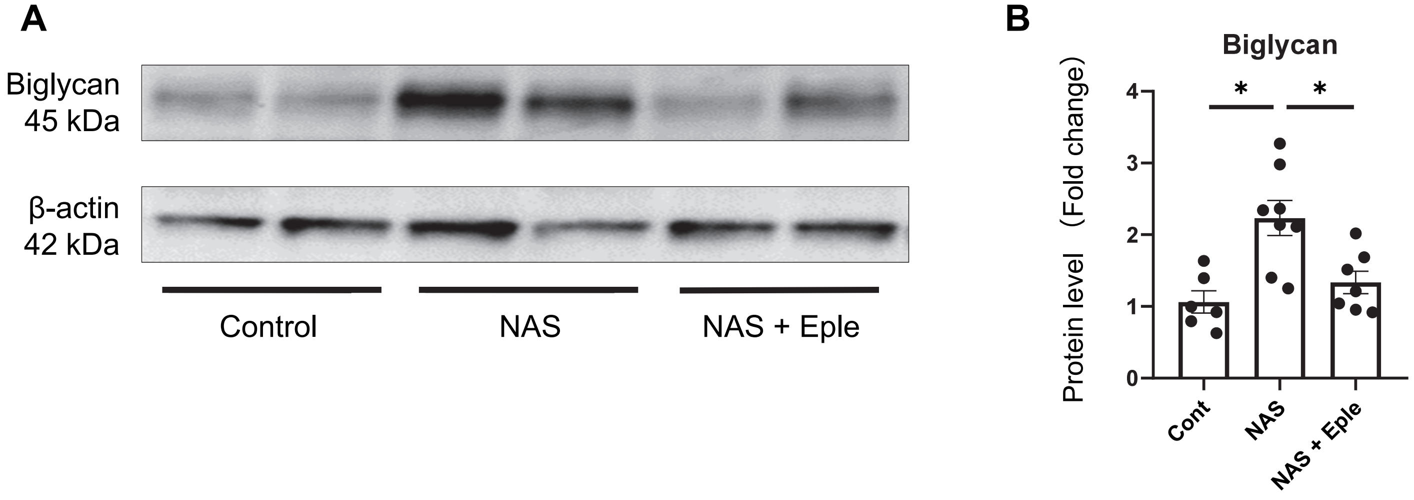

Figure S2. Eplerenone blunts the NAS-induced increase of biglycan expression in the kidney cortex.

Representative immunoblots of biglycan and β-actin. The box plots show the individual data normalized to the level of β-actin. One-way ANOVA was used for statistical analysis, n = 6–8. *$P < 0.05$. NAS, uninephrectomy/aldosterone/salt; Eple, eplerenone.

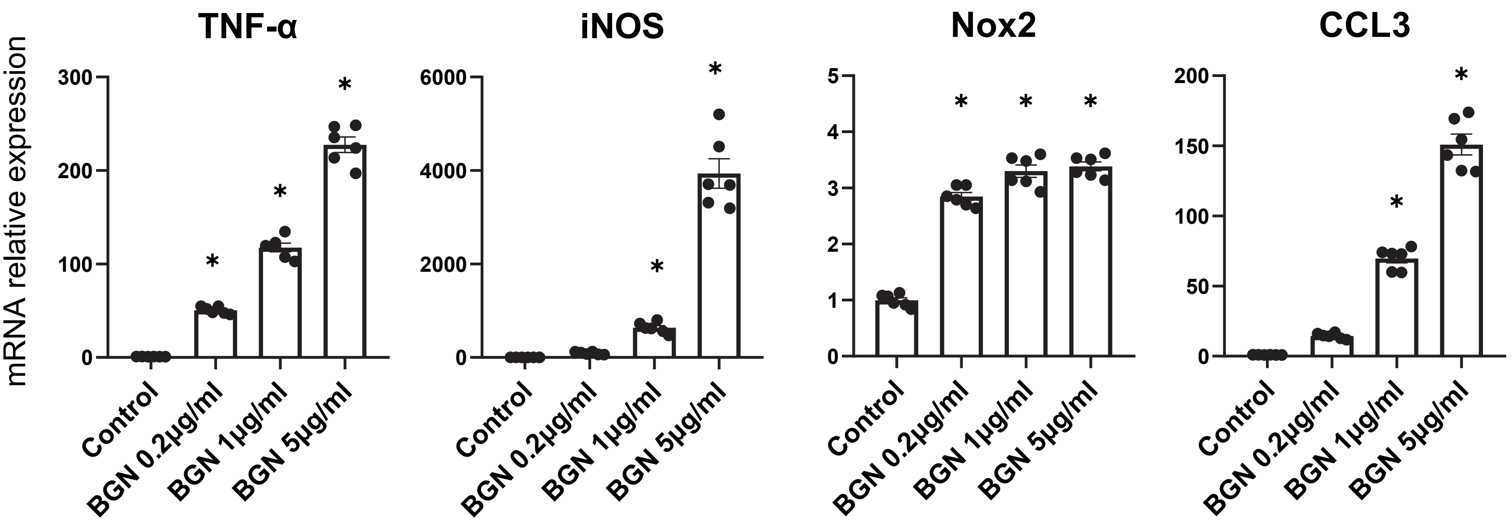

Figure S3. Dose-dependency of the biglycan effect on macrophages.
Isolated peritoneal macrophages were treated with the indicated concentrations of biglycan (0.2-5.0 μg/ml) for 4 h. One-way ANOVA was used for statistical analysis, n = 6–8. *P < 0.05. TNF-α, tumor necrosis factor-α; iNOS, inducible nitric oxide synthase; Nox2, NADPH oxidase 2; CCL3, C-C motif chemokine ligand 3; BGN, biglycan.

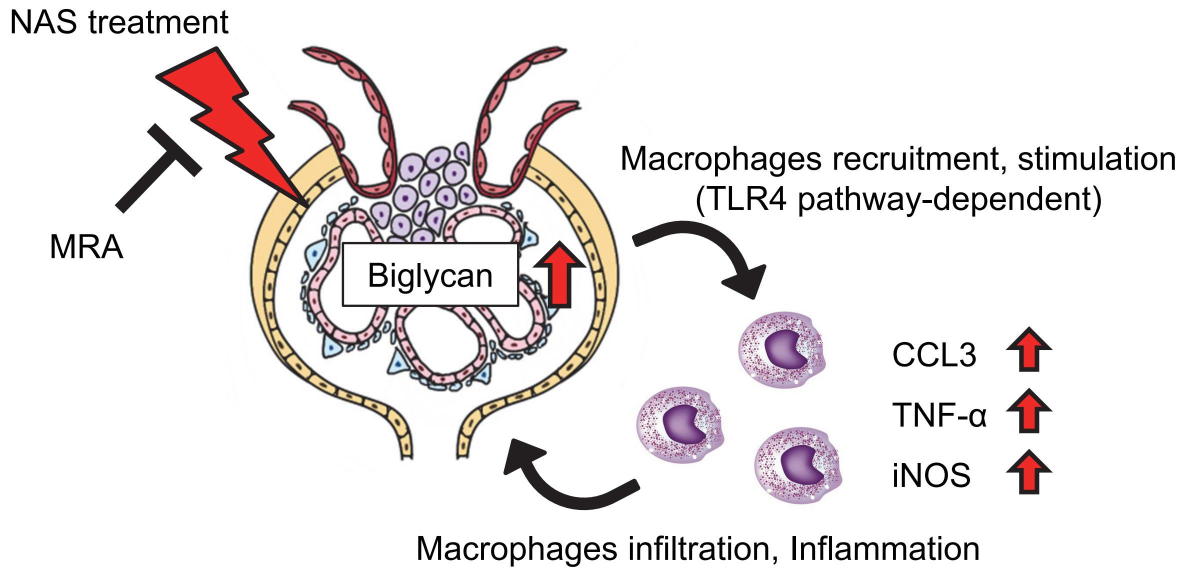

Figure S4. Proposed mechanism of biglycan-induced glomerular injury

Table S1. Primers used for gene expression analyses

Gene product name		Primer Sequence
GAPDH	Forward	5'-AATGGTGAAGGTCGGTGTG-3'
	Reverse	5'-GAAGATGGTGATGGGCTTCC-3'
Collagen type 1a1	Forward	5'-GCTCCTCTTAGGGGCCACT-3'
	Reverse	5'-CCACGTCTCACCATTGGGG-3'
Collagen type 3a1	Forward	5'-CTGGAGCCCCTGGACTAATAG-3'
	Reverse	5'-GCCCATTTGCACCAGGTTCT-3'
TGF-β1	Forward	5'-TGCGCTTGCAGAGATTAAAA-3'
	Reverse	5'-CTGCCGTACAACTCCAGTGA-3'
Lumican	Forward	5'-TCGAGCTTGATCTCTCCTAT -3'
	Reverse	5'-TGGTCCCAGGATCTTACAGAA-3'
Biglycan	Forward	5'-CTACGCCCTGGTCTTGGTAA-3'
	Reverse	5'-ACTTTGCGGATACGGTTGTC-3'
Syndecan-1	Forward	5'-ACTTCACCTTTGAAACATCTGGG-3'
	Reverse	5'-CATCCGGTACAGCATGAAAGC-3'
Syndecan-4	Forward	5'-CATCTTTGAGAGAACTGAGGTCTTG-3'
	Reverse	5'-CCTTCTTCTTCATGCGGTACA-3'
CD68	Forward	5'-ACAAGGGACACTTCGGGCCA-3'
	Reverse	5'-GTCGTCTGCGGGTGATGCAG-3'
CD86	Forward	5'-AGCAGACGCGTAAGAGTGGCT-3'
	Reverse	5'-CATGGTGCATCTGGGGTCCATC-3'
CD206	Forward	5'-CCACAGCATTGAGGAGTTTG-3'
	Reverse	5'-ACAGCTCATCATTTGGCTCA-3'
CCL2	Forward	5'-GGCTGGAGAGCTACAAGAGG-3'
	Reverse	5'-TCTTGAGCTTGGTGACAAAAAC-3'
CXCL2	Forward	5'-ATCCAGAGCTTGAGTGTGACGC-3'
	Reverse	5'-AAGGCAAACTTTTTGACCGCC-3'
CCL5	Forward	5'-GCCCTCACCATCATCCTCACT-3'
	Reverse	5'-GGCGGTTCCTTCGAGTGACA-3'
CXCL13	Forward	5'-ATATGTGTGAATCCTCGTGCCA-3'
	Reverse	5'-GGGAGTTGAAGACAGACTTTTGC-3'
CCL3	Forward	5'-TTCTCTGTACCATGACACTCTGC-3'
	Reverse	5'-CGTGGAATCTTCCGGCTGTAG-3'
TNF-α	Forward	5'-CATCTTCTCAAAATTCGAGTGACAA-3'
	Reverse	5'-TGGGAGTAGACAAGGTACAACCC-3'
iNOS	Forward	5'-GCCAAGGCCAAACACAGCATAC-3'
	Reverse	5'-TGGCCACCTTGTTCAGCTACG-3'
Nox2	Forward	5'-CGCCCTTTGCCTCCATTCTC-3'
	Reverse	5'-CCTTTCCTGCATCTGGGTCTCC-3'

GAPDH, glyceraldehyde 3-phosphate dehydrogenase; TGF-β1, transforming growth factor β1; CCL2, C-C motif chemokine ligand 2; CCL3, C-C motif chemokine ligand 3; CCL5, C-C motif chemokine ligand 5; CXCL2, C-X-C motif chemokine ligand 2; CXCL13, C-X-C motif chemokine ligand 13; TNF-α, tumor necrosis factor-α; iNOS, inducible nitric oxide synthase; Nox2, NADPH oxidase 2.
